# Presence of *Salmonella* AvrA in colorectal tumor and its precursor lesions in mouse intestine and human specimens

**DOI:** 10.18632/oncotarget.19052

**Published:** 2017-07-06

**Authors:** Rong Lu, Maarten Bosland, Yinglin Xia, Yong-guo Zhang, Ikuko Kato, Jun Sun

**Affiliations:** ^1^ Division of Gastroenterology and Hepatology, Department of Medicine, University of Illinois at Chicago, Chicago, IL, USA; ^2^ Department of Pathology, University of Illinois at Chicago, Chicago, IL, USA; ^3^ Division of Academic Internal Medicine and Geriatrics, Department of Medicine, University of Illinois at Chicago, Chicago, IL, USA; ^4^ Department of Oncology and Pathology, Wayne State University School of Medicine, Detroit, MI, USA

**Keywords:** colorectal cancer, inflammation, infection, IBD, Salmonella

## Abstract

Evidence directly supporting an association between *Salmonella* infection and colorectal cancer in human subjects is sparse. It has been well recognized that *Salmonella* infection increases the risk of gallbladder cancer. AvrA, a bacterial protein from *Salmonella* enterica, plays a crucial role in establishing chronic infection. To our knowledge, the presence of the bacterial AvrA has never been studied in human samples. Here, we demonstrated the presence and cellular localization of AvrA in inflamed, colorectal tumor and its precursor lesions, using both animal experimental infection models and human clinical specimens. We performed a newly developed AvrA serological assay and to determine the presence of anti-*Salmonella* AvrA antibody in chronic infected mouse serum samples. Further, we tested the presence of AvrA gene in healthy human fecal samples, in order to advance etiological studies of *Salmonella* AvrA in human population. Our study suggests a potential role of this bacterial protein in human colorectal cancer. Moreover, our new serological assay may serve a useful tool to identify individuals at increased risk for colorectal cancer.

## INTRODUCTION

The gastrointestinal tract is a natural habitat for a dynamic and highly competitive microbial community, which constantly contacts with the intestinal epithelial cells. Although growing evidence suggests a potential role of microbes in the development of colorectal cancer [1, 2], evidence to support a direct link of intestinal bacteria to human sporadic colorectal cancer is still limited. *Salmonella* *enterica* is a Gram-negative, facultative anaerobe and an intracellular pathogen to both humans and animals, posing a major public health concern worldwide. It is estimated that more than 1 million people in the US acquire *Salmonella* infection annually as a foodborne illness [3]. Seroepidemiologic studies have revealed that non-typhoid *Salmonella* infection is much higher (˜600 times) than actually reported [4], ranging from 56 per 1000 person-years in Finland to 547 in Poland [5] and rising over years [6]. It has been well recognized long-standing *Salmonella* infection increases the risk of gallbladder cancer [7–9]. However, evidence directly supporting an association between *Salmonella* infection and colorectal cancer in human subjects is sparse.

A common aspect of infection-related cancer is the induction of chronic inflammation, which may promote DNA damage, cell proliferation and migration, through various mechanisms including epigenetic modifications [10, 11]. Many pathogens, such as *Salmonella*, use type three secretion system (T3SS) to inject numerous virulence factors that induce strong proinflammatory reactions [12]. Whereas most bacteria induce inflammation in the host, some pathogenic bacteria have also evolved the abilities to temper the inflammatory response to create a suitable niche for their survival and proliferation in the host, not killing the host or host cells they infected. AvrA, a T3SS effector protein from *Salmonella* enterica, plays a crucial role in establishing chronic infection [13–15]. AvrA is a 33 kDa protein and a close homologue to a family of acetyltransferases expressed in several enteric pathogens, including YopJ/P in *Yersinia pseudotuberculosis* and VopA in *Vibrio parahemalyticus* [15]. AvrA exerts anti-inflammatory activities through inhibition of NF-κB and JNK pathways, resulting in reduced secretion of inflammatory mediators [16]. Furthermore, this JNK inhibition leads to suppression of apoptosis particularly in the context of proinflammatory enteropathogenic Salmonellosis [13–15], and thus to prolonged bacterial intracellular survival.

We have revealed that *Salmonella* AvrA possesses deubiquitination properties [12], leading to activation of the β-catenin pathway. Subsequent studies using mouse models have revealed that infection with AvrA-expressing *Salmonella* increased Wnt and total β-catenin expression, Wnt/β-catenin transcriptional activity and the numbers of stem cells and of proliferative cells in infected intestinal mucosa, underscoring the role of AvrA in stem cell maintenance [17]. In the carcinogen azoxymethane (AOM)/ inflammatory agent dextran sodium sulphate (DSS) colon cancer model [18], colorectal tumor incidence indeed significantly increased in the AvrA^+^ *Salmonella* infected mice, compared with mice without bacterial gavage or infected with AvrA^−^ *Salmonella* [18]. In our previous studies, we confirmed chronic colonization of AvrA-expressing *Salmonella*, but we have not examined the localization of AvrA protein in infected intestine and tumor.

To our knowledge, the presence of the bacterial virulence AvrA has never been studied in human samples. The primary aim of this study is to demonstrate the presence and intra-cellular localization of AvrA in inflamed, colorectal tumor and its precursor lesions in both animal experimental infection models and human clinical specimens. The secondary aims were to test performance of newly developed AvrA serological assay and to determine the presence of anti-*Salmonella* AvrA antibody in chronic infected mouse serum samples. Further, we tested the presence of *AvrA* gene in healthy human fecal samples, in order to advance etiological studies of *Salmonella* AvrA in human population.

## RESULTS

### Detectable anti-AvrA antibody in serum of mice 10 weeks post AvrA-positive *Salmonella* infection

**Figure 1:**
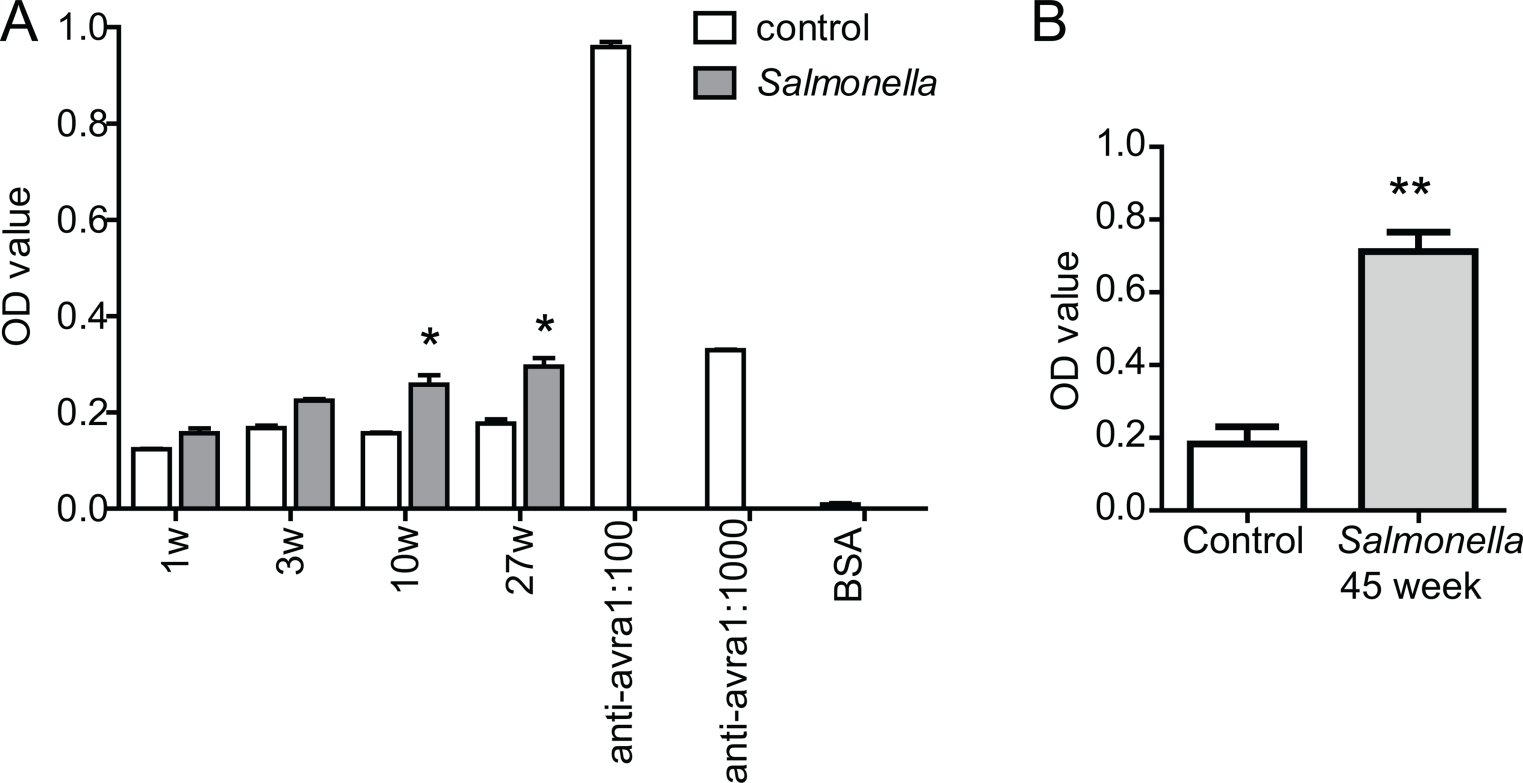
Elisa measurement of anti-AvrA protein in mouse serum (**A**) Mice were infected with *Salmonella* mutant strains PhoP^C^AvrA^−^/AvrA^+^ by oral gavage. Serum anti-AvrA were measured at 1, 3, 10 and 27 weeks postinfection. (**B**) Anti-AvrA protein of mouse serum in colon cancer model. Serum anti-AvrA antibody was measured at in the AOM/DSS mice 45 weeks post *Salmonella* infection. We used anti-AvrA antibody as a positive control and 1% BSA as a negative control. *P < 0.05, **P < 0.01, *n* = 3, by Student's *t* test.

We developed an ELISA measurement to test the existence of anti-AvrA antibody in mice post *Salmonella* infection. First, combinations of different dilutions of antigen and antibodies were tested for titration. Then the assay was applied to mouse serum from the long-term experimental *Salmonella* infection model (Figure 1). We used anti-AvrA antibody as the positive control and 1% BSA as a negative control in these experiments. As shown in the Figure 1, we were able to detect the significant increased AvrA antibody in mouse serum 10 weeks post AvrA-positive *Salmonella* infection. We could also see the significantly increased Optical density (OD) value of anti-AvrA antibody in the mice post infection 27 week in inflammation model (Figure 1A). Using samples from the *Salmonella*-infected mice in AOM/DSS colon cancer model [18], we were able to detect significantly high value of anti-AvrA antibody in mouse serum 45 week post *Salmonella*-infected (Figure 1B).

### Location of AvrA in *Salmonella* infected mouse colon

**Figure 2:**
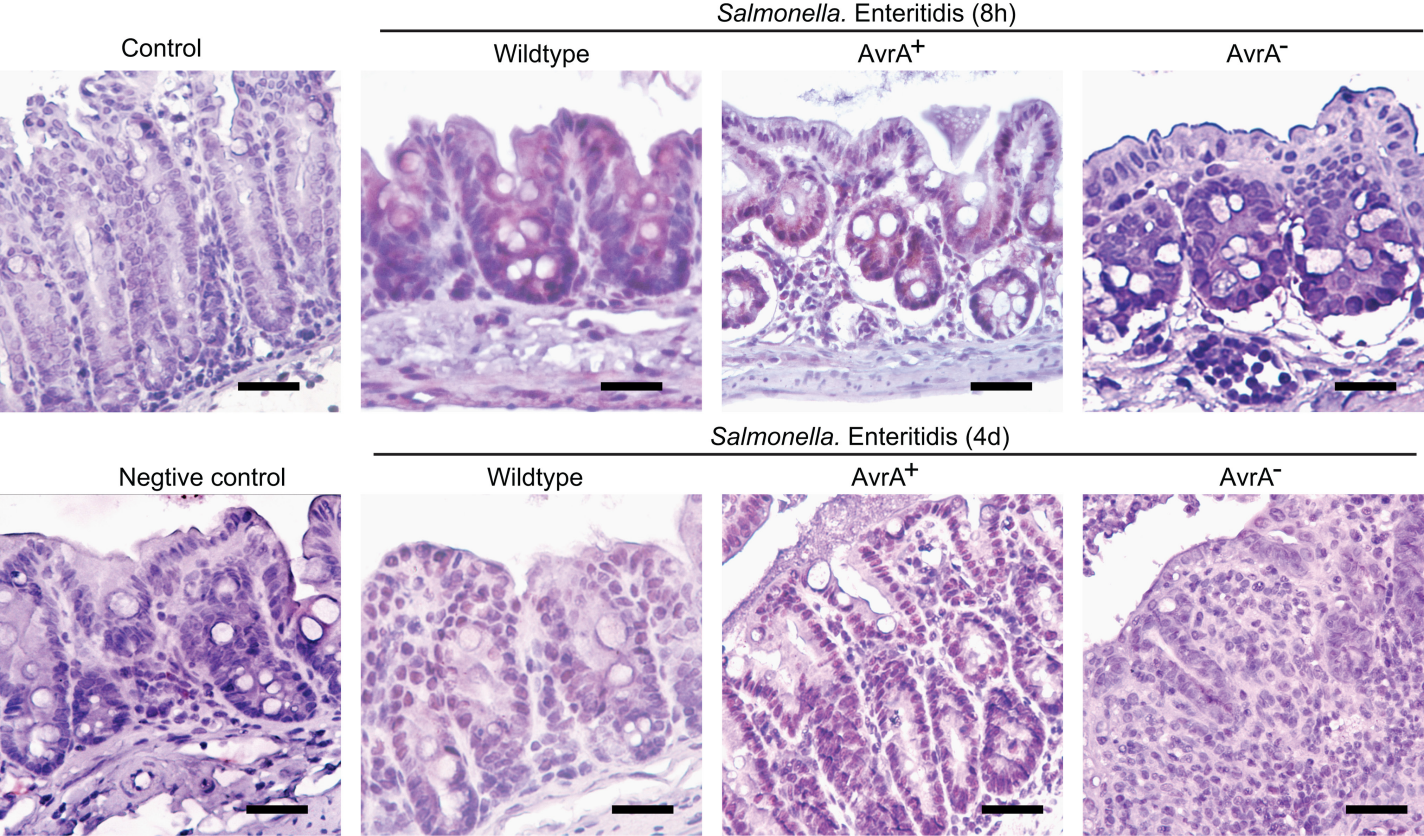
IHC of AvrA staining in mouse intestine infected with *Salmonella* Enteritidis Mice were infected with wild-type *Salmonella* Enteritidis C50336, AvrA mutant S.E-AvrA^−^ and the complemented strain S.E-AvrA^+^ [43] by oral gavage. Immunostaining of AvrA in the mouse colon tissue 8 hour and 4 day post-*Salmonella* infection. *n* = 3 per group.

The immunohistochemistry (IHC) was used to examine the location of AvrA in *Salmonella* ser. Enteritids infected mouse colon post infection 8 hours and 4 days. We have samples infected with Salmonella strains with or without AvrA. The results showed the nuclear staining of AvrA (brown color) in epithelial cells in the *S*. Enteritids wild-type group (Figure 2). Strong nuclear staining of many epithelial cells and some sub-epithelial stroma were found in the *S*. Enteritids AvrA^+^ group. In contract, no positive AvrA staining was found in the *S*. Enteritids AvrA^−/−^ group (Figure 2).

**Figure 3:**
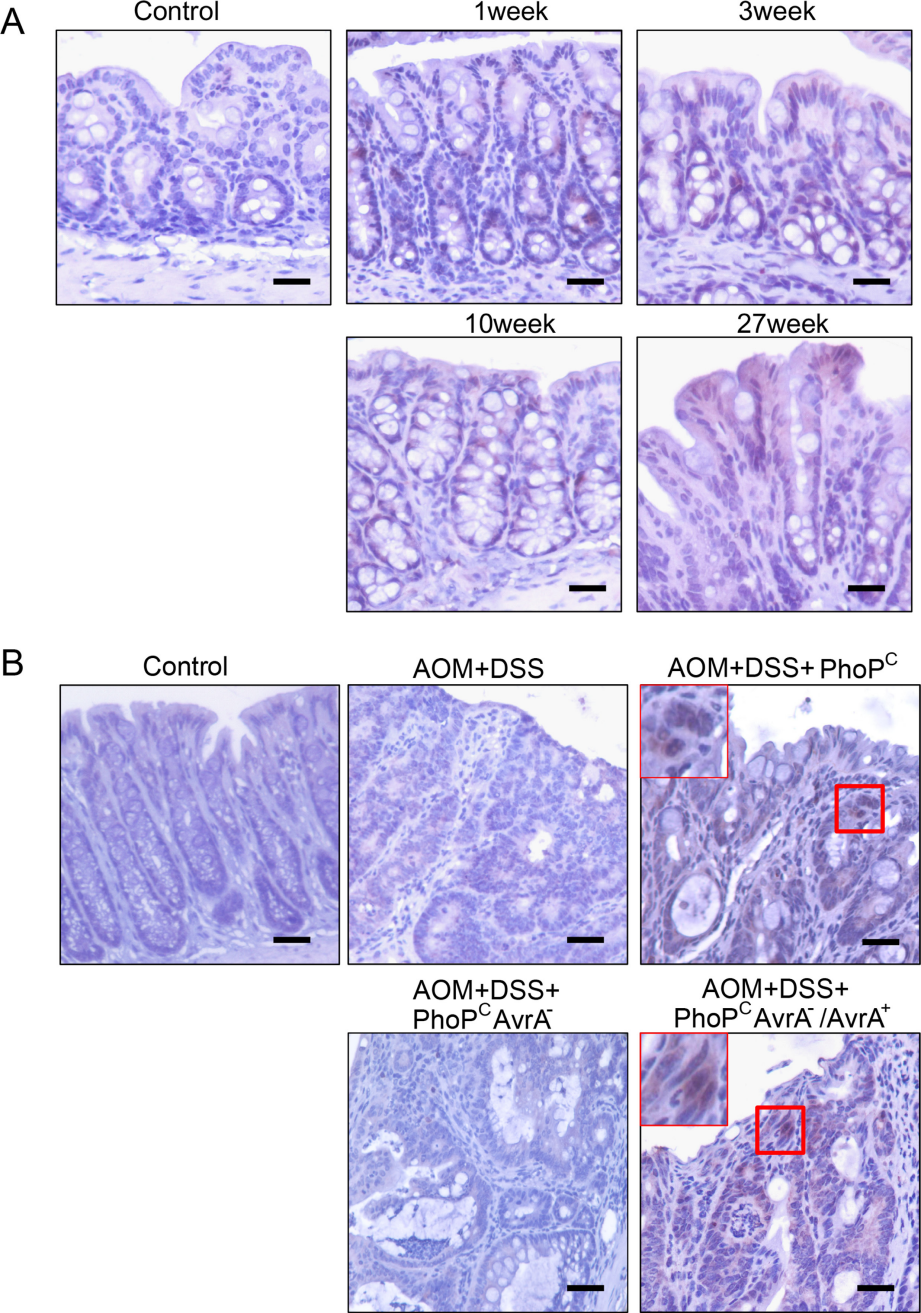
IHC of AvrA staining in mouse intestine with chronic infection (**A**) In the *Salmonella*-chronically infected mouse colon, we were able to detect the positive AvrA staining in colon 1, 3, 10, and 27 week post infection. These data indicate the persistent expression of AvrA in the *Salmonella* infected colon. (**B**) AvrA in mouse intestine with chronic infection and colon cancer. Immunostaining of AvrA in colon of mouse carcinogenesis model with *Salmonella* expressing AvrA in the mouse colon cancer model. Scale bar = 500 um. *n* = 3 per group.

In the *Salmonella*-chronically infected mouse colon, we were able to detect the positive AvrA staining in colon 1, 3, 10, and 27 week post infection (Figure 3A). These data indicate the persistent expression of AvrA in the *Salmonella* infected colon.

### Presence of AvrA in colon of mouse carcinogenesis model with *Salmonella* expressing AvrA

In the chemical carcinogenesis AOM/DSS model, colorectal tumor incidence indeed markedly increased in the AvrA^+^ *Salmonella* infected mice, compared with mice without bacterial gavage or infected with AvrA^-^ *Salmonella* [18]. We found that strong nuclear staining of AvrA in the colon with tumor from AOM+DSS+ PhoP^C^AvrA^−^/AvrA^+^ mice 45 weeks post infection (Figure 3B). In contrast, no AvrA expression in tumors from mice infected AOM+DSS+ PhoP^C^AvrA^−^ group and the AOM+DSS mice without *Salmonella* infection. Taken together, we confirmed persistent AvrA staining in the colon infected with the AvrA-expressing *Salmonella* strains (Figure 3B).

### AvrA expression in human inflammatory bowel diseases (IBD) and colon samples

We then examined the presence of AvrA protein in IBD and colorectal tumor tissue in human clinical samples. We found positive AvrA staining, including the nuclear staining of AvrA, in intestinal epithelial cells of the patients with IBD (Crohn's disease and ulcerative colitis). Among the 27 inflammation category cores, there were only 6 formally diagnosed IBD cases (5 Crohn's disease and 1 ulcerative colitis), the rest was annotated as chronic inflammation. Regardless, these cores present inflammatory cell infiltration. But we do not have detailed information as to whether IBD was in active stage. When we divided this group to IBD and other inflammation, mean normalized AvrA staining score was slightly higher in IBD (2.26 ± 0.35SE) than in the others (1.91 ± 0.19SE), but the difference was not statistically significant.

**Figure 4:**
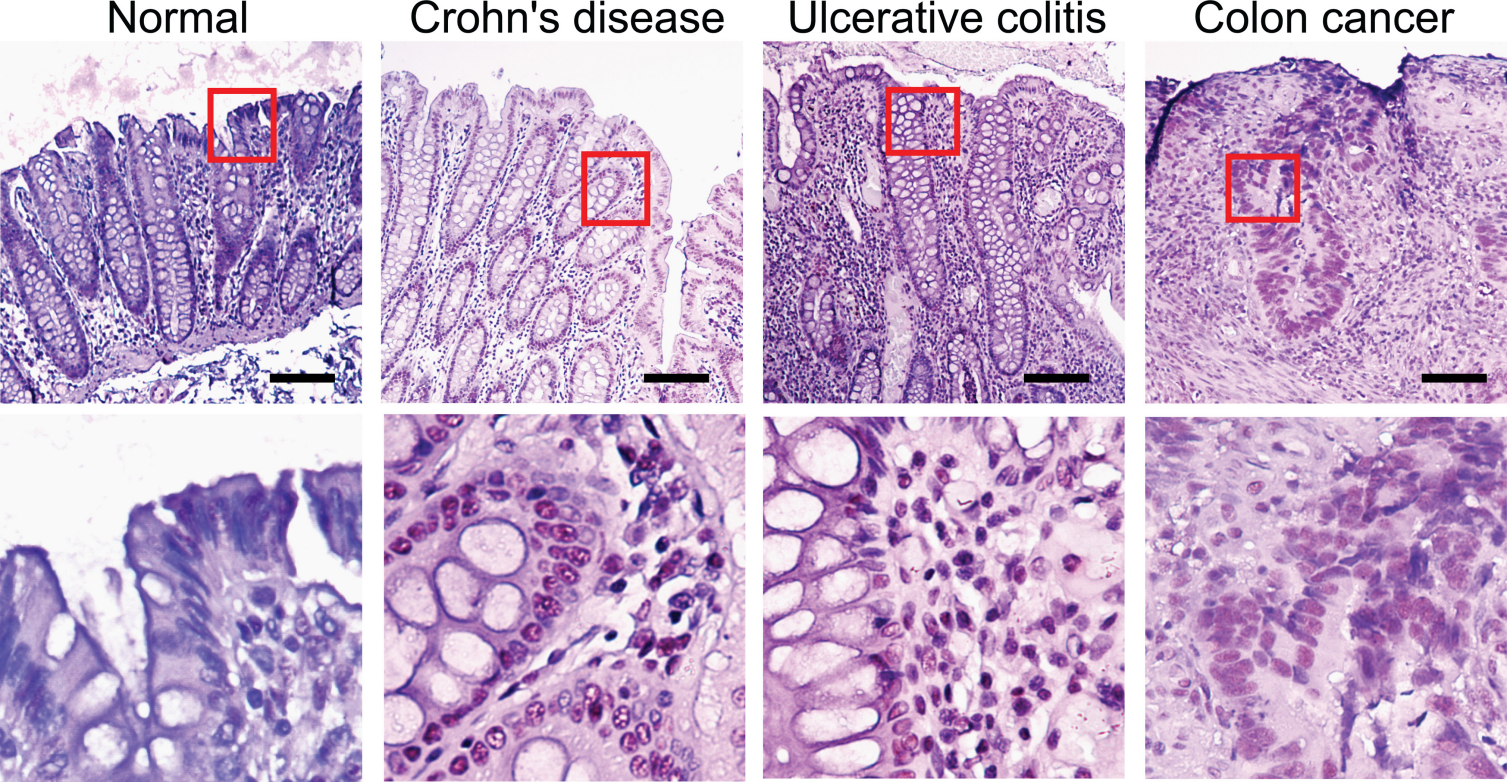
IHC of AvrA staining in human tissue of IBD and colon cancer Immunostaining of AvrA in human normal, IBD and colon tumor tissue. Scale bar = 100 um.

The immunohistochemistry (IHC) data from human normal mucosa and cancer tissue clearly show dense red staining of AvrA in cancer tissue, including nuclei (Figure 4), whereas AvrA staining is negative in normal colon (low and high power).

### AvrA staining in human TMA samples

The three TMA chosen for this study included variable numbers of colorectal histologies in different proportions as shown in Table 1. Approximately a half from unique colorectal cancer patients, divided into primary tumor (*n* = 48), adjacent normal mucosa (*n* = 13) and metastasized lymph nodes (*n* = 14). Another half consisted of benign lesions (34 adenoma and 27 inflammation) and normal mucosa without any colorectal pathologies (*n* = 19). As reported in Table 2, after normalization each array showed the identical mean staining score. Compared with normal mucosa without any colorectal pathology, cancer adjacent mucosa had a statistically significantly higher mean normalized staining score (*P* = 0.018), while primary tumors themselves exhibited a significantly lower mean score (*P* = 0.013). Benign lesions and lymph nodes showed equivalent staining to normal mucosa (Table 3). Interstingly, we also identified the nuclear staining of AvrA in intestinal epithelial cells of the colon cancer patients.

**Table 1:** Distribution of colorectal pathologies of tissue cores included from 3 tissue micro arrays

Pathology	TMA slides				
		CO808-036	CO809a-C051	BC05002a-E068	Total
Colon Cancer	*N*	20	10	18	48
	%	34.48	17.54	45.0	
Node metastasis	*N*	8	0	6	14
	%	13.79	0	15.0	
Cancer adjacent	*N*	9	0	4	13
	%	15.52	0	10	
Adenoma	*N*	7	26	1	34
	%	12.07	45.61	2.5	
Inflammation	*N*	10	12	5	27
	%	17.24	21.05	12.5	
Normal mucosa	*N*	4	9	6	19
	%	6.9	15.79	15.0	
Total		58	57	40	155

**Table 2:** Raw and normalized AvrA staining scores for three TMA slides

TMA name	*N*	AvrA scores	Mean	Median	Quartile 1	Quartile 3
CO808-036	58	Raw	10.103	10.00	6.00	15.00
		Normalized	1.771	1.77	1.07	2.57
CO809a-C051	57	Raw	2.158	1.00	0.00	2.00
		Normalized	1.771	1.66	0.66	2.36
BC05002a-E068	40	Raw	1.175	1.00	0.00	1.00
		Normalized	1.771	2.10	0.87	2.10

**Table 3:** Mean normalized staining scores by colorectal pathology

Pathology	*N*	Normalized sore mean	Standard Error	*P*-values*
Colon cancer	48	1.372	0.125	0.0126
Node Metastasis	14	1.880	0.231	0.7853
Cancer Adjacent	13	2.719	0.240	0.0177
Adenoma	34	1.649	0.148	0.2070
Inflammation	27	1.988	0.166	0.9218
Normal mucosa	19	1.963	0.198	–

*Difference from normal mucosa

### Presence of AvrA gene in healthy human fecal samples

**Figure 5:**
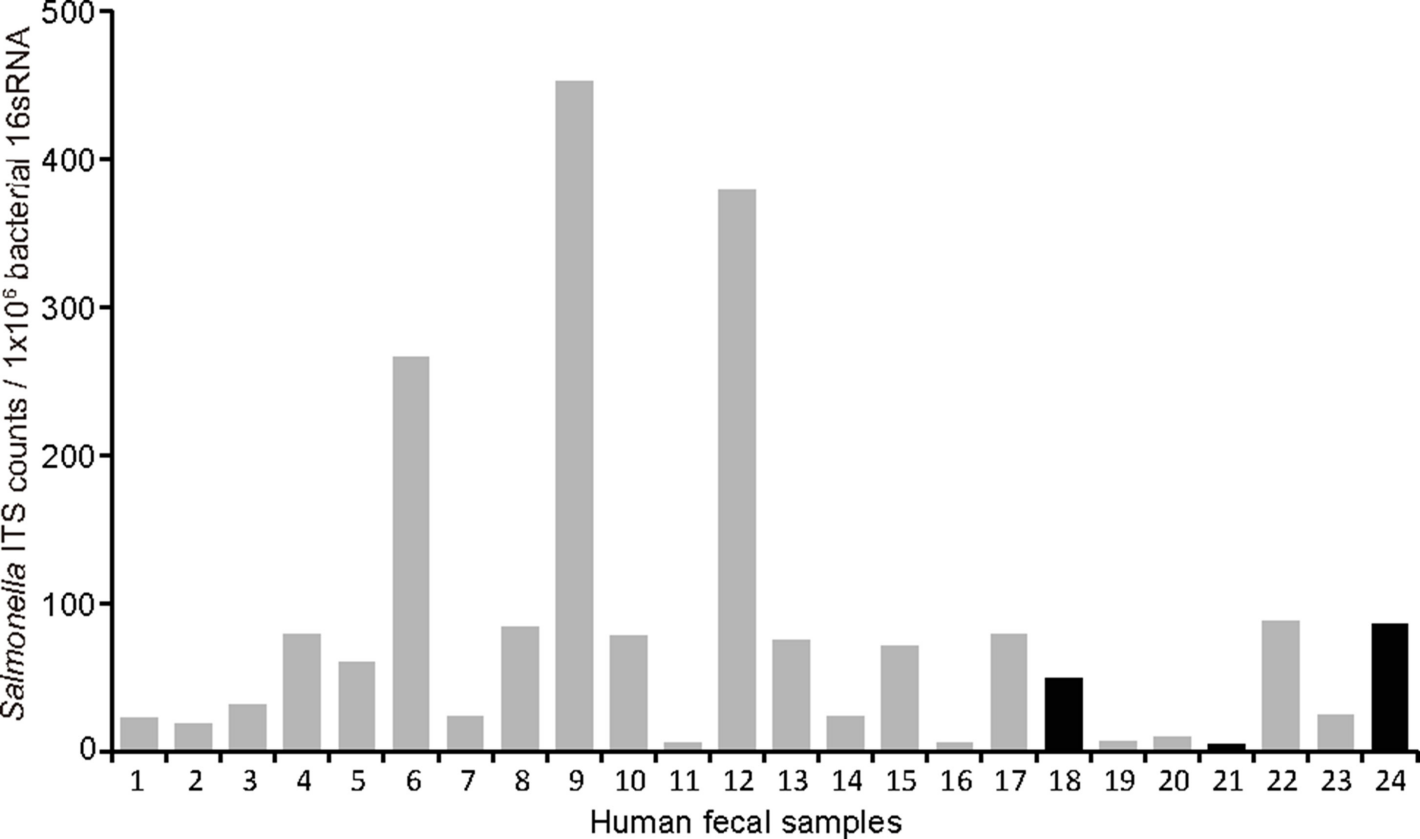
Amplification for *Salmonella* AvrA in human fecal samples Human DNA samples (*n* = 24) were amplified using real-time PCR. We applied primers for universal bacterial 16sRNA, *Salmonella* 16S-23S internal transcribed spacer (ITS), as well as *Salmonella* AvrA. We found little variation in overall bacterial 16S RNA, relative *Salmonella* counts, assessed by ITS over universal bacterial 16S RNA, varied markedly from a sample to a sample. All of these samples except three (black/dark bars) also exhibited amplification for *Salmonella* AvrA.

We found little variation in overall bacterial 16S RNA among 24 human fecal samples, but as shown in Figure 5, relative *Salmonella counts*, assessed by *Salmonella* 16S-23S internal transcribed spacer (ITS) over universal bacterial 16S RNA, varied markedly from a sample to a sample. All of these samples except three (black/dark bars, No. 18, 21, and 24) also exhibited amplification for *Salmonella* AvrA. These data are consistent with high prevalence of antibodies against *Salmonella* flagellin found in Metropolitan Detroit population [19].

## DISCUSSION

In our current study, we, for the first time, have demonstrated the presence of *Salmonella* AvrA protein in colorectal mucosa from mice with experimental infection and human clinical specimens. Also, we showed the detectable anti-AvrA-antibody in mouse serum post *Salmonella* infection by ELISA. Using IHC, we found the distribution AvrA in *Salmonella* infected mouse colon and in *Salmonella*-infected mice with colon cancer. Furthermore, we reported the presence of AvrA protein in inflammatory bowel disease and colorectal tumor tissue in human clinical samples. Human fecal samples exhibited amplification for *Salmonella* AvrA. Our study suggests a novel role of bacterial protein associated with colon cancer.

Our IHC data of anti-AvrA showed that AvrA is more commonly found in adjacent mucosa than primary tumors. It is not necessarily unexpected that pathogens are less commonly detected in cancer tissue than adjacent normal mucosa. The presence of bacteria may no longer be necessary once carcinogenic pathways are activated, as seen in the case of gastric cancer where *H Pylori* is less frequently detected in advanced premalignant lesions than in less advanced lesions [20]. Furthermore alteration of cell surface glycosylation is often found in various cancers [21] and it also influences intestinal microbial composition [22]. Recently an enzyme catalyzing glycosylation, B4GALNT2, has been associated with increased susceptibility to *Salmonella* infection [23], while its expression has been reported to be downregulated in colon cancer compared with normal mucosa [24]. These findings well corroborate our observation that more AvrA is found in adjacent mucosa than in primary tumors.

*AvrA* gene is present in most *Salmonella* enterica isolates from humans and animals. This is particularly the case for non-typhoid *Salmonella* as its prevalence gene was reported to be 98–100% (514/523 and185/185) [25, 26], while it was absent in typhoid strains. On the contrary, AvrA protein expression is known to vary markedly with clinical presentation. AvrA protein is not often produced by clinical isolates from systemic disease, but it is often detectable in those from limited enteritis [27]. Our data of AvrA presence in the colon of chronically infected mice indicate the persistent function of bacterial proteins in the host. It is now clear that expression of AvrA is regulated in a post-transcriptional manner and that mRNA transcription takes place constitutively in all AvrA-positive strains [28], as AvrA remains silent when cloned into *E. coli* [29]. In fact, a number of proteobacterial pathogens have evolved global regulation systems of posttranscriptional gene expression for their various virulence factors to be responsive to changes in the environment and to flourish in specialized host niches and *Salmonella enterica* is equipped with Csr (carbon storage regulator) system, consisting of a small RNA binding protein (CrsA) and non-coding RNAs (CsrB/CsrC) [30]. CsrA binding to the 5′ untranslated and/or early coding regions of mRNAs alters mRNA translation, turnover and/or transcript elongation, leading to either decay or stabilization of specific mRNA targets, while CsrB/CsrC containing multiple CsrA binding sites bind and sequester CsrA, thereby antagonizing CsrA activities [30, 31]. Kerrinnes *et al*. [28] demonstrated that overexpression of either CsrA or CsrB shut down AvrA expression, but that constitutional CsrA expression was required for AvrA expression. These posttranscriptional controls of AvrA highlight the need to study its protein levels rather than nuclei acid levels.

The location of AvrA is shown in both cytosol and nuclei. Previous studies discovered deubiquitinase and acetyltransferase properties of AvrA leading to upregulation of the beta-catenin signaling pathway and modifying p53 activities [14, 15, 18], which are critical in initiation and progression of colorectal cancer. Ubiquitination and deubiquitination are in fact major host targets of human oncoviruses, including HPV, KSHV, EBV, HTLV1 and adenovirus [32]. Known host targets of AvrA O (threonine)- and N^ε^(lysine)-acetylation include MAPKK [13] and p53 [12, 33]. Acetylation of critical amino acid residues in MAPKK blocks MAPKK phosphorylation activities, thus inhibits downstream c-Jun N-terminal kinase and NF-κB signaling pathways [12]. This may in turn compensatedly activate STAT3 signaling and inflammation, as shown in mouse model of AvrA induced colorectal carcinogenesis [34]. On the other hand, p53 acetylation has been shown to increase its stability and transcriptional activities [35]. Separately from the role of AvrA effector in intestinal epithelial cells, *Salmonella* SopB, another effector from the same pathogenicity island, transforms primed epithelial cells into M cells to promote host colonization and invasion [36] The autocrine activation of RelB-expressing FAE enterocytes by up-regulated RANKL/RANK further guides the EMT-regulating transcription factor Slug that marks epithelial transdifferentiation into M cells. Based on these observations and our current data, AvrA may play a novel role through posttranslational modifications associated with colon cancer. It is still not known how AvrA moves to the nuclei and interacts with host genes/proteins.

We previously reported that antibody against *Salmonella flagellin* was higher in colorectal cancer and pre-cancer cases than in controls in two distinct populations in US and the Netherlands and that smoking and dietary intake (i.e., iron) is one of the mediating factors, suggesting a possible link of *Salmonella* to colorectal cancer [19]. Yet, information is still limited regarding frequencies of sustained *Salmonella* infection after initial acquisition in the population. It is not clear that how AvrA plays its role in the human colon cancer. In our previous study, we found that AvrA can activate the beta-catenin pathway in human colon cancer cells, such as Caco2-BBE and HCT 116 [12, 14]. Our unpublished data also indicate the overlapping of nuclear staining of AvrA and *β-catenin in samples from human colon cancer*. We will investigate whether bacterial AvrA exploits host posttranslational modification of key proteins, such as β-catenin, in oncogenic pathways to promote cell proliferation and colorectal tumorigenesis in the future studies.

To our knowledge, this is the first to report the presence of *Salmonella* AvrA protein in human tissue specimens as well as that antibodies against AvrA are in fact detectable in infected hosts. The latter finding will prompt studies to investigate whether human subjects infected with AvrA-expressing *Salmonella* have an increased risk of developing colorectal cancer using pre-diagnostic sera from prospective cohorts.

## MATERIALS AND METHODS

### Human samples

Human fecal DNA samples used for this study were a part of deidentified samples from the previous study where stool specimens were collected in RNA later from non-cancer volunteers in Metropolitan Detroit. Details concerning the parent study have been reported elsewhere [37–40]. The human tissue microarray (TMAs) used in our studied were purchased from US Biomax, Inc. (Biomax, Derwood, MD), and stained as described previously [14, 18, 41, 42].

### Animals and ethics statement

C57BL/6 mice (Female, 6–8 weeks old) were obtained from the Jackson Laboratory (Jackson Laboratory, Bar Harbor, ME, USA). All animal work was approved by University of Rochester and University of Illinois at Chicago Committee on Animal Resources. Euthanasia was accomplished via sodium pentobarbital (100 mg per kg body weight) I.P., followed by cervical dislocation. All methods were carried out in accordance with the approved guidelines by Committees on Animal Resources.

### Bacterial strains and growth condition

*Salmonella* strains used in this study included *Salmonella* mutant strains PhoP^C^AvrA^−^/AvrA^+^, wild-type *Salmonella* Enteritidis C50336, AvrA mutant S.E-AvrA^-^ and the complemented strain S.E-AvrA^+^ (Table 4 [43]). Bacterial cultures were prepared by inoculating 10 ml of Luria–Bertani broth with 0.01 ml of a stationary-phase culture followed by overnight incubation (> 18 h) at 37°C, as previously described [41, 44].

**Table 4:** *Salmonella* strains used in the current study

Strains	Characteristics	References
S.E-WT	*Salmonella* Enteritidis wild type CMCC(B)50336	[43]
S.E- AvrA^-^	C50336 AvrA-deficient mutant	[43]
S.E- AvrA^+^	C50336 AvrA-deficient mutant carrying pBR322-AvrA	[43]
PhoP^C^	Non-pathogenic complex regulator mutant derived from wild-type SL14028	[18, 41]
PhoP^C^AvrA^-^	AvrA^-^ mutation derived from PhoP^C^	[18, 41]
PhoP^C^AvrA^-^/AvrA^+^	PhoP^C^ AvrA^-^ with complemented plasmid encoding AvrA	[18, 41]

### Real-time PCR of bacteria 16sRNA and *Salmonella*

Real-time PCR was used to amplify universal bacterial 16sRNA, *Salmonella* 16S-23S internal transcribed spacer, as well as *Salmonella* AvrA. Total DNA was extracted from human fecal Qiagen Stool Kit (Qiagen, Hilden, Germany and DNA was then subjected to real-time PCR (SYBR Green PCR kit, BioRad) with primers (Table 5). Percent expression was calculated as the ratio of the normalized value of each sample relative to that of the corresponding control group. All real-time PCR reactions were performed in triplicate.

**Table 5:** Primers for real-time PCR

Name	sequence 5′–3′
Univ bacteria 16s F	TCCTACGGGAGGCAGCAGT
Univ bacteria 16s R	GGACTACCAGGGTATCTAATCCTGTT
*Salmonella* ITS F	TATGCCCCATCGTGTAGTCAGAAC
*Salmonella* ITS R	TGCGGCTGGATCACCTCCTT
*Salmonella* AvrA F	GAATGGAAGGCGTTGAATCTGC
*Salmonella* AvrA R	GTTGTGCGCCTTGAGTATGTTTGTAA

### *Salmonella*-infected mouse model

Animal experiments were performed using specific pathogen-free female C57BL/6 mice. Water and food were withdrawn 4 h before oral gavage with 7.5 mg/mouse of streptomycin (100 ml of sterile solution). Afterwards, animals were supplied with water and food ad libitum. Twenty hours after streptomycin treatment, water and food were withdrawn again for 4 h before the mice were infected with 1 × 10^6^ colony-forming units of *Salmonella* (100-ml suspension in HBSS (Hank's Balanced Salt Solution) or treated with sterile HBSS (control) by oral gavage as previously described [41, 44]. After *Salmonella* gavage, tissue samples were collected at 8h and 4 days for short term model and at 1, 3, 10, 27 weeks for *Salmonella*-chronically infected model.

### *Salmonella* infected colon cancer mouse model

Animal experiments were performed by using specific pathogen–free female C57BL/6 mice (Taconic) that were 6–7 weeks old, as previously described [45] Water and food were withdrawn 4 h before oral gavage with 7.5 mg/mouse of streptomycin (100 μl of sterile solution). Afterwards, animals were supplied with water and food *ad libitum*. Twenty hours after streptomycin treatment, water and food were withdrawn again for four hours before the mice were infected with 1 × 10^6^ CFU of *S. typhimurium* (100-μl suspension in HBSS) or treated with sterile HBSS (control) by oral gavage, as previously described [46] After *Salmonella* gavage, AOM/DSS were administrated as follows: azoxymethane (AOM), 10 mg/kg body weight, intraperitoneal injection, 1% dextran sodium sulfate (DSS) in drinking water. At 45 weeks after *Salmonella* infection, tissue samples were collected.

### Immunohistochemistry

Intestinal tissues were freshly collected and embedded in paraffin wax after fixation with a 10% neutral buffered formalin. Immunohistochemistry was performed on paraffin-embedded sections (4 μm) of colons. After preparation of the slides as described previously [14, 18, 41, 42] slides were incubated in 3% hydrogen peroxide for 20 minutes at room temperature to block endogenous peroxidase activity, followed by incubation for 60 minutes in 2% BSA in PBS to reduce nonspecific background. The slides were incubated with anti-AvrA antibody at 4°C overnight. Samples were then incubated with goat anti-rabbit antibody (Jackson ImmunoResearch, West Grove, PA, USA) for 1 hour at room temperature. Anti-AvrA antibody was custom-made as previously described) [33].

### *Salmonella* anti-AvrA antibody enzyme-linked immunosorbent assay

*Salmonella* AvrA antibody in mouse serum was measured using an AvrA antibody enzyme-linked immunosorbent assay. The 96-well plate was coated with AvrA protein (1 μg/ml) 4^o^ overnight. *Salmonella* AvrA protein was purified in Sun lab [33]. The coated plate was blocked with 1% BSA 4^°^ overnight. Mouse serum (100 ul per well, 1:5 dilution) were added to the well for 2 h in 37^°^ incubator. Following complete washing, add 1:300 diluted HRP-conjugated Goat anti-mouse antibody (Bio-rad, Hercules, CA) to each well for 1 h in 37^°^. Following complete washing, KPL SureBlue peroxidase substrate was added. Measure the absorbance of each well at 450 nm. We used anti-AvrA antibody (1:100&1:1000) as positive control and 1% BSA as negative control. The anti-AvrA antibody was custom-made and used in our previous studies for Western blots [47]. The 15 amino acid [36] peptide CGEEPFLPSDKADRY was designed based on the AvrA sequence aa#216–230 (GenBank accession no. AE008830).

### Statistical analysis

Generally, descriptive statistics for continuous variables were expressed as mean ± standard deviation (SD), categorical variables were presented as frequency and proportion. For AvrA staining scores, mean, median, and quartile were reported. All statistical tests were two sided. *P* values of 0.05 or less were considered statistically significant.

Data were expressed as mean ± SD in Elisa assay (Figure 1). Differences between Salmonella and control groups were analyzed by Student's *t* test with GraphPad Prism 5.

The TMAs were scored by a single well trained veterinarian pathologist who has more than 30 years of experience (Dr. Maarten Bosland, Professor of Department of Pathology, UIC). IHC of AvrA staining was initially assessed as a product of staining intensity (0, 1, 2, 3, 4 = no staining, minimal, slight, moderate, marked intensity) and percentage of cells stained (1, 2, 3, 4, 5 = few scattered (occasional cells), several scattered cells, focal (one or a few areas or cells), multifocal (several areas of cells), diffuse (most cells), ranging from 0 to 20. Out of the 168 cores scored on three TMA slides, hyperplasia (*n* = 8) was excluded due to too small sample size as a single diagnostic category. Further we took only one of the cores with the highest staining score for the 5 paired samples derived from same patients present within the same TMA slides. As a result, the final analytical sample consisted of 155 unique cores. The compassion of the results of the three TMA slides revealed substantial staining variability among slides, specifically generally much stronger staining in the 1st slide, much weaker in the 2nd and even weaker in the 3rd. Thus, to combine results from three TMA slides, we calculated normalized staining scores based on relative rankings of individual core staining within each slide using method described by Blom [48], which generates normalized scores centered at 0. To make all scores positive, each score was added to the lowest score among all TMAs. As a result, the final normalized scores ranged from 0 to 4. These results were summarized on Table 2. Then, analysis of variance was used to test differences in mean normalized scores across 5 different colorectal pathologies (inflammation, adenoma, cancer adjacent mucosa, colorectal cancer and metastasized lymph nodes) from normal mucosa. The statistical analyses were conducted by SAS version 9.4 (SAS Institute Inc., Cary, NC, USA).
